# Multidimensional Drivers of Phytoplankton Assembly in a Karst Reservoir: Seasonal Dynamics and Regulatory Implications

**DOI:** 10.3390/plants15071024

**Published:** 2026-03-26

**Authors:** Zhongxiu Yuan, Mengshu Han, Lan Chen, Yan Chen, Jing Xiao, Qian Chen, Qiuhua Li, Yongxia Liu

**Affiliations:** 1 Key Laboratory for Information System of Mountainous Area and Protection of Ecological Environment of Guizhou Province, Guizhou Normal University, Guiyang 550001, China; 18708659007@163.com (Z.Y.); jing-xiao2022@gznu.edu.cn (J.X.); gznuchenqian@126.com (Q.C.); 2 Guizhou Key Laboratory of Advanced Computing, Guiyang 550001, China; hanmengshu112@163.com (M.H.); cy@gznu.edu.cn (Y.C.); 3 Guizhou International Science & Technology Cooperative Base of Aquatic Ecology Research, Guiyang 550001, China; 4 School of Cyber Science and Technology, Guizhou Normal University, Guiyang 550001, China; 5 College of Foreign Languages, Guizhou Normal University, Guiyang 550001, China; cindymoon829@126.com; 6 Guizhou Provincial Center for Ecological and Environmental Monitoring, Guiyang 550081, China

**Keywords:** Baihua reservoir, ecological network analysis, multi-dimensional drivers, phytoplankton community assembly, targeted ecological regulation

## Abstract

Baihua Reservoir, a typical large waterbody in the karst region of southwestern China and an essential drinking water source, is characterized by a high carbonate buffering capacity that profoundly shapes the structure and function of its phytoplankton community. This study systematically elucidates the multi-dimensional driving mechanisms underlying seasonal phytoplankton community assembly in karst reservoirs by integrating multiple analytical models—including the Neutral Community Model, β-diversity decomposition, co-occurrence network analysis, XGBoost-SHAP machine learning, and Partial Least Squares Path Modeling—based on monthly sampling at five sites from 2020 to 2024. The results revealed that: (1) Stochastic processes dominated community assembly across all four seasons, while deterministic processes played a crucial role in local species turnover. (2) The co-occurrence network structure showed significant seasonal dynamics, with the composition of keystone species adaptively shifting in response to changing environmental conditions. (3) The key environmental factors influencing the phytoplankton community exhibited clear seasonal patterns, primarily pH, NH_3_-N, and COD_Mn_ in spring; water temperature, COD_Mn_, and NH_3_-N in summer; TN, TP, and pH in autumn; and pH, water temperature, and DO in winter. To support the sustainable management of karst reservoirs, we propose seasonally differentiated strategies derived from our phytoplankton community analysis: target COD_Mn_ reduction in spring and summer, focus on TN and TP load control in autumn, prioritize water column stability in winter, and maintain hydrological connectivity and pH monitoring year-round. This approach enhances phytoplankton community stability, safeguards drinking water safety, and provides a targeted management model for similar reservoir ecosystems globally.

## 1. Introduction

As primary producers in aquatic ecosystems, phytoplankton form the material and energy basis of the food web and play irreplaceable ecological functions in driving nutrient cycling, regulating dissolved oxygen balance in water bodies, and supporting secondary productivity [1,2]. Due to its short lifecycle and rapid generational turnover, population dynamics are highly sensitive to environmental fluctuations [3,4]. Small changes in environmental factors such as light, temperature, nutrient concentration, and hydrological conditions may trigger the community structure and dominant population replacement of phytoplankton [5]. Therefore, phytoplankton are not only the core components for maintaining the functions of aquatic ecosystems, but their community composition and succession characteristics are also often used as important indicators for evaluating the nutritional status and ecological health of water bodies [6,7]. Understanding what drives these community patterns is therefore a fundamental question in aquatic ecology. Typically, community assembly is shaped by the interplay of biotic and abiotic factors, priority effects, and dispersal processes [8]. Under the framework of niche theory, which posits that species possess distinct ecological niches. phytoplankton community assemblies are generally controlled by deterministic processes, primarily environmental conditions (such as pH, temperature, and nutrients) and interspecific interactions (including competition, predation, and mutualism) [9]. However, niche theory has been challenged by neutral theory, which posits that all species are functionally equivalent and that community dynamics are governed by stochastic processes, such as birth, death, dispersal, and ecological drift, rather than by differences in competitive ability [10]. A substantial body of studies has revealed that deterministic and stochastic processes are not mutually exclusive; rather, they both play significant and simultaneous roles in community assembly, collectively shaping biodiversity and species composition [11,12]. Globally, research across diverse aquatic ecosystems—from temperate lakes in North America to tropical reservoirs in Southeast Asia and coastal seas in Europe—has consistently demonstrated that the relative importance of these processes varies with environmental context, spatial scale, and seasonal dynamics [13,14,15,16]. Disentangling the relative roles of deterministic versus stochastic processes in community assembly has been a major focus in community ecology [17].

In aquatic ecosystems, coexisting species interact through the exchange of matter, energy, and information, such as in forms of competition, facilitation, and mutualism, forming a complex ecological network [18]. The Microbial Co-occurrence Network serves as a key analytical method for studying the structure and interactions of biological communities [19]. The construction of a phytoplankton ecological network allows us to elucidate inter-species relationships, pinpoint keystone species, and thereby shed light on the mechanisms governing their coexistence and interaction, ultimately enhancing our understanding of ecosystem organization. Previous studies have shown that key factors driving phytoplankton communities vary seasonally [20]. Therefore, the mechanisms of community assembly and the underlying drivers of co-occurrence networks may also vary by season. However, the assembly mechanisms of phytoplankton communities and the stability of co-occurrence networks in reservoir ecosystems remains understudied, particularly for karst reservoirs, which are particular in environmental conditions.

As a typical karst drinking water reservoir, Baihua Reservoir confronts distinctive challenges including high carbonate buffering capacity and seasonal nutrient pulses from agricultural runoff. These environmental conditions may significantly affect phytoplankton community characteristics and assembly processes [21,22]. Nevertheless, no existing study has integrated co-occurrence networks with machine learning approaches to investigate its community dynamics [23,24]. This study employed an integrated analytical framework to systematically elucidate the community assembly mechanisms and multidimensional driving factors behind seasonal phytoplankton dynamics in the Baihua Reservoir. First, the Neutral Community Model and β-diversity decomposition were applied to investigate community assembly from the perspectives of stochastic and deterministic processes, respectively. Second, co-occurrence network analysis was used to clarify species interactions and community dynamics. Then, an interpretable XGBoost-SHAP machine learning framework was implemented to quantify the impact thresholds of various environmental factors on phytoplankton abundance variations and to explain their relative contributions. Finally, Partial Least Squares Path Modeling was utilized to reveal the direct and indirect interactions among these factors, thereby providing a scientific basis for the management of Baihua Reservoir.

## 2. Results

### 2.1. Phytoplankton Community Structure

**Figure 1:**
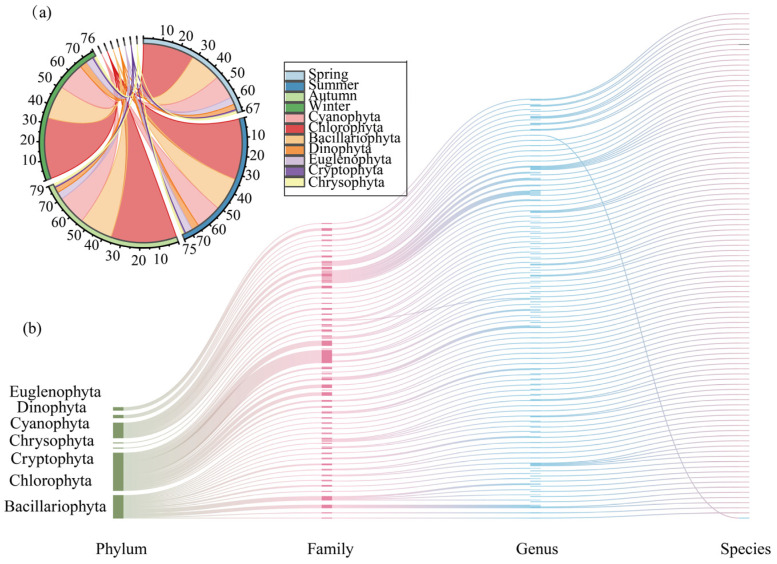
Phytoplankton Community Composition in the Baihua Reservoir (**a**); Seasonal composition of phytoplankton community in Baihua Reservoir (**b**).

A total of 99 phytoplankton species, belonging to 7 phyla, were identified in the Baihua Reservoir during the study period (Figure 1a). Chlorophyta was the most diverse group with 44 species, followed by Bacillariophyta (27 species) and Cyanobacteria (18 species). The remaining phyla included Dinophyta (4 species), Euglenophyta (4 species), Cryptophyta (1 species), and Chrysophyta (1 species). The phytoplankton community in the reservoir was characterized as a chlorophyta-diatom type. Seasonal investigation revealed 67 species (7 phyla) in spring, 75 species (7 phyla) in summer, 79 species (7 phyla) in autumn, and 76 species (7 phyla) in winter (Figure 1b).

**Figure 2:**
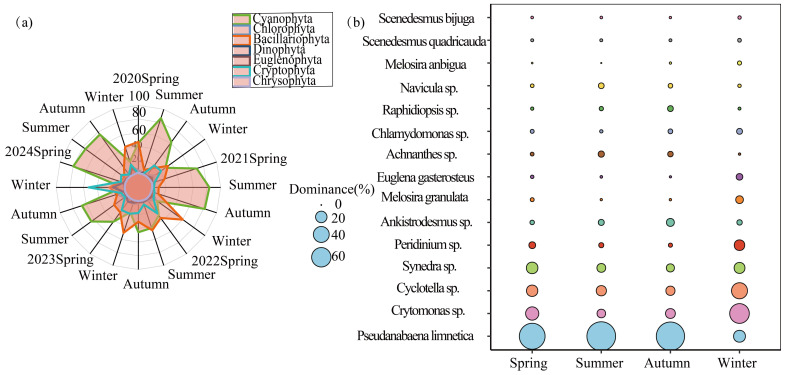
Seasonal Relative Abundance (**a**) and Dominant Species Dominance (**b**) of Phytoplankton in the Baihua Reservoir from 2020 to 2024. Note: (**a**) illustrates the seasonal variations in the phylum-level relative abundance of phytoplankton. Different colors represent different phytoplankton phyla, and the height of the radar chart indicates the proportion of each phylum in the total abundance. (**b**) presents a bubble plot of the dominance values for the top 15 dominant species. The vertical axis lists the names of the dominant species, while the horizontal axis represents the four seasons (spring, summer, autumn, and winter). The size of each bubble reflects the dominance value (Y value) of the corresponding species in that season, with larger bubbles indicating higher dominance.

Analysis of phytoplankton abundance in the Baihua Reservoir from 2020 to 2024 showed that it ranged from 0.11 × 10^4^ to 5005 × 10^4^ cells/L (Figure 2a). The average abundances across the four seasons were 11,416 × 10^4^ cells/L in spring, 27,184 × 10^4^ cells/L in summer, 15,015 × 10^4^ cells/L in autumn, and 3124 × 10^4^ cells/L in winter. The highest species richness was observed in autumn, while the highest average abundance occurred in summer. Overall, Cyanobacteria contributed the largest proportion to the total phytoplankton abundance in all seasons. The top 15 dominant species were calculated and visualized in a bubble plot (Figure 2b). The results revealed that *Pseudanabaena limnetica*, *Cryptophyta, Cyclotella*, and *Synedra* were highly dominant throughout all seasons. *Pseudanabaena limnetica* was the most dominant species in spring, summer, and autumn, whereas *Cryptophyta* became the predominant species in winter.

### 2.2. Phytoplankton Community’s Assembly

The parameters R^2^ and Nm from the neutral model are used to evaluate the influences of stochastic and deterministic processes. R^2^ measures the model’s fit, with a high value indicating community structure is largely stochastic. Nm, the product of metacommunity size and migration rate, indicates dispersal capacity, where a high value leads to homogeneous species distribution. A community assembly dominated by stochastic processes is inferred when both R^2^ and Nm are high, whereas dominance by deterministic processes is attributed to low values for both [25]. The solid line in the figure represents the best-fit line calculated based on neutral theory, while the dashed line typically represents the confidence intervals of the fitted model.

**Figure 3:**
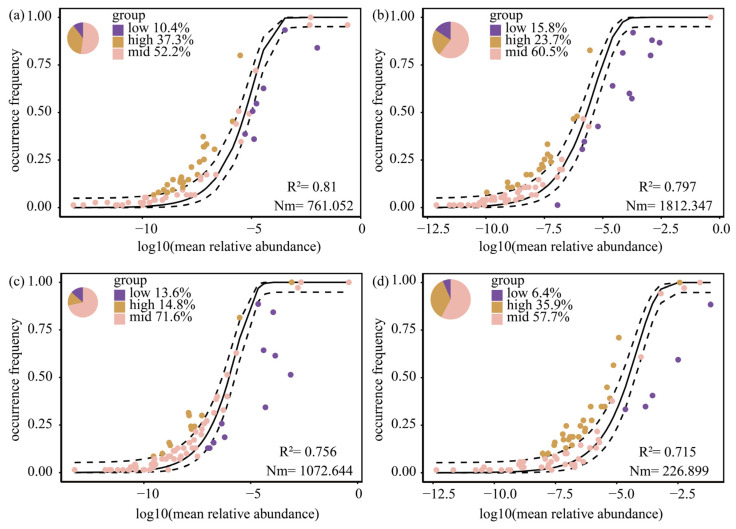
Neutral Community Model of Phytoplankton by Season: (**a**) Spring, (**b**) Summer, (**c**) Autumn, (**d**) Winter.

The Neutral Community Model analysis revealed that stochastic processes dominated phytoplankton community assembly across all four seasons in the Baihua Reservoir (Figure 3). The fit of the neutral model (R^2^) was highest in spring (0.810), followed by summer (0.797), autumn (0.756), and winter (0.715), indicating that the relative contribution of stochastic processes gradually decreased from spring to winter. Species dispersal capacity, represented by the migration parameter Nm, peaked in summer (24.52), followed by autumn (19.78), spring (17.34), and winter (10.21), suggesting that hydrological connectivity and species exchange were strongest during the summer season. These results indicate a seasonal shift in the balance between stochastic and deterministic assembly processes, with stochasticity playing a greater role in spring and summer, while deterministic processes gained relative importance in autumn and winter.

**Figure 4:**
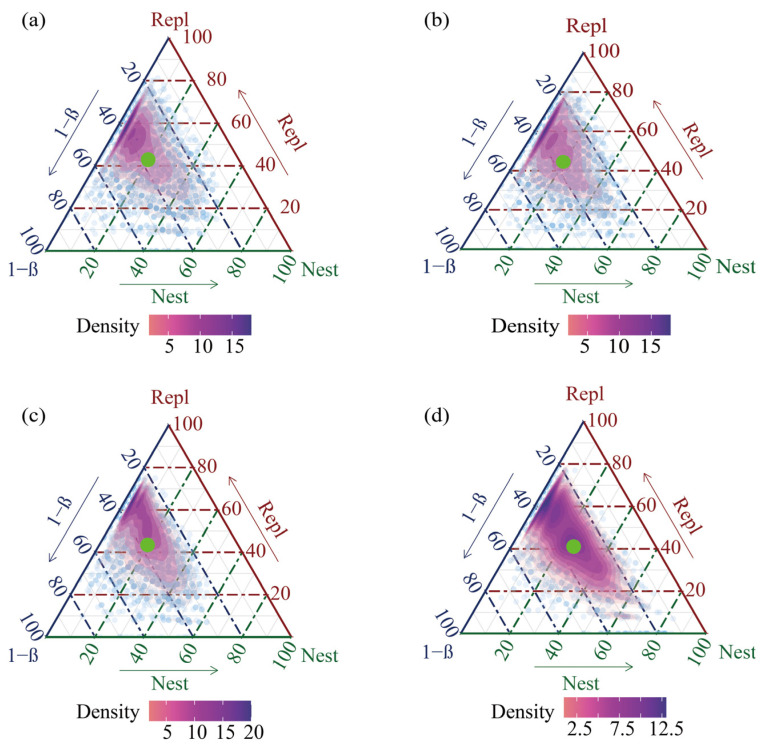
Partitioning of Phytoplankton Community β-diversity in the Baihua Reservoir across Four Seasons: (**a**) Spring, (**b**) Summer, (**c**) Autumn, (**d**) Winter.

The neutral community model did not fully explain the variations in the phytoplankton community, indicating the concurrent existence of other assembly processes and mechanisms, such as environmental selection and species interactions. Therefore, β-diversity decomposition was employed for further investigation. β-diversity between paired samples was quantified with both the Sørensen and Jaccard indices. These overall indices were further decomposed into the turnover (Repl) and nestedness (Nest) components [26,27]. Community similarity is denoted by (1-β). A high proportion of the turnover component, which reflects niche processes, signifies that community differences arise primarily from species replacement along environmental gradients, thereby indicating the dominance of deterministic processes [28]. The nestedness-resultant component pinpoints the role of dispersal limitation and stochasticity in shaping community differences. Its high contribution underscores the dominance of stochastic processes, where species are randomly lost or gained [29]. Community similarity is quantified as (1-β). Graphically, the distribution density of communities is visualized by colored regions (color intensity corresponds to sample density), and the mean decomposition pattern for each group is indicated by a green point. The ternary plot visualization of β-diversity partitioning revealed that the relative proportions of the turnover and nestedness components in the phytoplankton community varied seasonally (Figure 4). This demonstrates a shifting balance between deterministic and stochastic assembly processes across different seasons.

Note: β-diversity between paired samples was quantified using the Sørensen index and decomposed into turnover (Repl) and nestedness (Nest) components. The ternary plots visualize the relative proportions of community similarity (1-β), turnover (Repl), and nestedness (Nest) for each seasonal group. Each dot represents a pairwise comparison between samples. The colored regions indicate the distribution density of communities, with color intensity corresponding to sample density (darker colors represent higher density). The green point in each plot marks the mean decomposition pattern for that season, providing a summary of the central tendency. A high contribution of the turnover component indicates dominance of deterministic processes (species replacement along environmental gradients), while a high contribution of the nestedness component suggests dominance of stochastic processes (species loss or gain due to dispersal limitation and random events). The results reveal seasonal shifts in the balance between deterministic and stochastic assembly processes.

As a supplement to the variation unexplained by environmental and spatial factors in community assembly, co-occurrence network analysis was used to elucidate the interrelationships among phytoplankton species and their critical importance in structuring the community [30]. The structure of the network serves as an indicator of community complexity and stability, as environmental fluctuations cause competitive relationships among species to vary, leading to phytoplankton community dynamics [31]. We applied co-occurrence network analysis to the phytoplankton community of the Baihua Reservoir, visualizing the network to elucidate species interactions [32]. In this study, we visualized the co-occurrence network of phytoplankton in the Baihua Reservoir to clarify their interrelationships. Network properties—including nodes, average clustering coefficient, average path length, and average degree—all exhibited spatiotemporal variations. These attributes are indicative of the complexity among species, transmission efficiency, and the efficiency of energy and information flow, as well as the sensitivity to external environmental disturbances.

Each node in the network represents a species, and its size is indicative of its relative importance within the network [33]. An edge represents the strength and direction of the correlation between two species. A thicker edge indicates a stronger correlation. The color of the edge is used to distinguish between positive and negative correlations. A positive correlation suggests potential complementary niches or mutualistic relationships between the species, while a negative correlation indicates possible competition or inhibitory interactions [34]. The average degree, indicating the density of inter-species interactions, influences network connectivity. The average path length governs the efficiency of information transfer, with shorter paths enabling faster propagation. The average clustering coefficient measures the degree to which species tend to cluster together, reflecting the modularity of the network. A high clustering coefficient indicates a highly modular structure, where species within the same module are tightly interconnected. This modular organization can enhance community stability by confining the impact of species loss or environmental disturbances to specific modules, thereby preventing cascading effects across the entire network. Conversely, a low clustering coefficient suggests a more randomly structured network with weaker internal cohesion, potentially making the community more susceptible to external perturbations [35].

**Figure 5:**
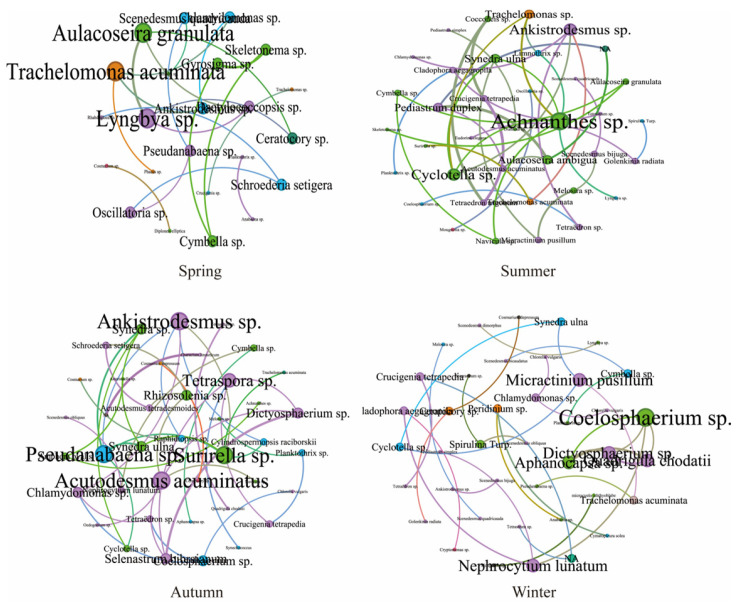
Seasonal dynamics of the phytoplankton co-occurrence network in Baihua Reservoir. Note: Nodes represent phytoplankton species, with the size reflecting their relative importance in the network; edges represent interspecific correlations, with the thickness indicating the strength of the correlation.

**Figure 6:**
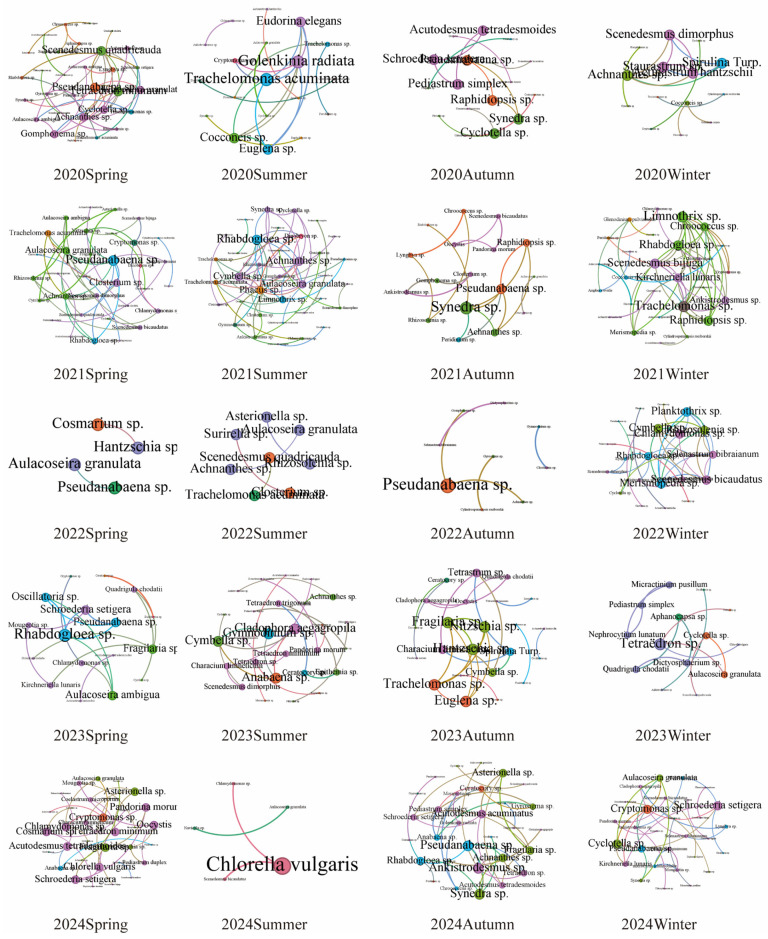
The interannual and seasonal variations of the co-occurrence network of phytoplankton.

Distinct seasonal dynamics were observed in the phytoplankton co-occurrence networks of the Baihua Reservoir (Figure 5, Table 1). Autumn and winter networks contained the most nodes, with autumn also showing the highest values for edges and average degree. Regarding interaction types, spring and winter networks were characterized solely by positive correlations, whereas summer and autumn exhibited a minor proportion of negative interactions. To illustrate the dynamics of the phytoplankton co-occurrence network in Baihua Reservoir, we visualized its seasonal variations over a five-year period (Figure 6). It can be clearly seen that the key species of phytoplankton community structure in Baihua Reservoir are different in different years and seasons, which proves that the phytoplankton community in Baihua Reservoir is a dynamic and adaptive complex system.

### 2.3. Multidimensional Drivers of Phytoplankton Community

**Figure 7:**
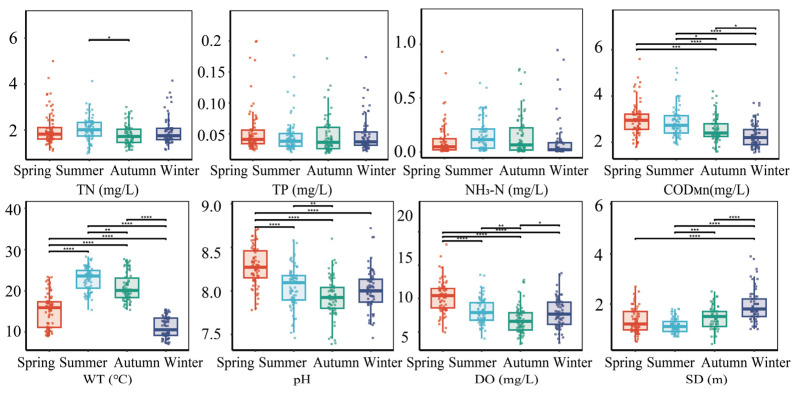
Seasonal Dynamics of Physicochemical Parameters in the Baihua Reservoir (* *p* < 0.05, ** *p* < 0.01, *** *p* < 0.001, **** *p* < 0.0001).

The seasonal variations in physicochemical parameters of Baihua Reservoir from 2020 to 2024 were assessed using box plots and one-way ANOVA (Figure 7). Except for total phosphorus (TP) and ammonia nitrogen (NH_3_–N), which exhibited no significant seasonal variation (*p* > 0.05), all other measured parameters displayed statistically significant seasonal changes (*n* = 289; *p* < 0.05).

Analysis of the monitored water quality and physical parameters in this study showed that all parameters exhibited certain fluctuations. The concentration of total nitrogen (TN) ranged from 0.98 to 5.00 mg/L with a mean of 1.95 mg/L. Total phosphorus (TP) varied between 0.02 and 0.20 mg/L, averaging 0.05 mg/L. Ammonia nitrogen (NH_3_–N) changed from 0.01 to 0.95 mg/L with a mean concentration of 0.13 mg/L. The permanganate index (COD_Mn_) ranged from 1.55 to 5.60 mg/L, with a mean of 2.68 mg/L. Meanwhile, water temperature (WT) varied from 7.10 to 28.30 °C, averaging 17.62 °C. The pH ranged from 7.39 to 8.72 (mean = 8.07), indicating an overall alkaline state of the water body. The dissolved oxygen (DO) concentration fluctuated between 3.20 and 18.10 mg/L, with a mean of 8.30 mg/L. The Secchi depth (SD) ranged from 0.40 to 3.90 m, with an average of 1.45 m.

**Figure 8:**
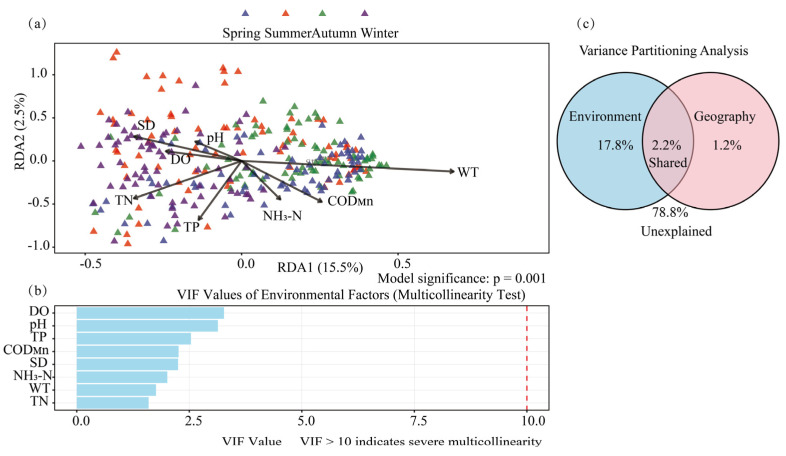
RDA of Environmental Factors and Phytoplankton Abundance. (**a**) RDA analysis of environmental factors and phytoplankton abundance; (**b**) VIF analysis among environmental variables; (**c**) VPA analysis of environmental and spatial factors on phytoplankton abundance variation.

Redundancy Analysis (RDA) was performed on the environmental factors and phytoplankton abundance data (Figure 8a). The results indicated that water temperature (WT), Secchi depth (SD), and the permanganate index (COD_Mn_) were identified as the key drivers of phytoplankton succession in the Baihua Reservoir. The Variance Inflation Factor (VIF) was used to assess multicollinearity among the environmental variables, where a higher VIF value indicates more severe collinearity. The calculated VIF values for each factor were all below 5 (a VIF > 10 suggests severe multicollinearity), indicating weak linear relationships among the predictors and a high independent explanatory power for each factor in the RDA model, thus confirming the reliability of the results (Figure 8b). A Variance Partitioning Analysis (VPA) was conducted to comprehensively evaluate the explanatory power of environmental and spatial factors on phytoplankton abundance variation (Figure 8c). The results showed that the explanatory power of environmental factors was higher than that of spatial factors, with a shared explanation of 2.2%, while 78.8% of the variation remained unexplained. This result is consistent with the stochasticity inferred from the Neutral Community Model.

The XGBoost-SHAP model is extensively used in multiple research fields to determine factor importance and interpret influence mechanisms. XGBoost is a decision-tree-based ensemble learning algorithm capable of modeling complex data relationships, specifically engineered to handle high-dimensional data and resolve nonlinear problems [36]. Leveraging the concept of Shapley values from cooperative game theory, SHAP (SHapley Additive exPlanations) provides a unified measure to interpret machine learning predictions. It works by quantifying the marginal contribution of each feature to the prediction outcome, offering a clear view of how individual features influence the model’s decisions.

**Figure 9:**
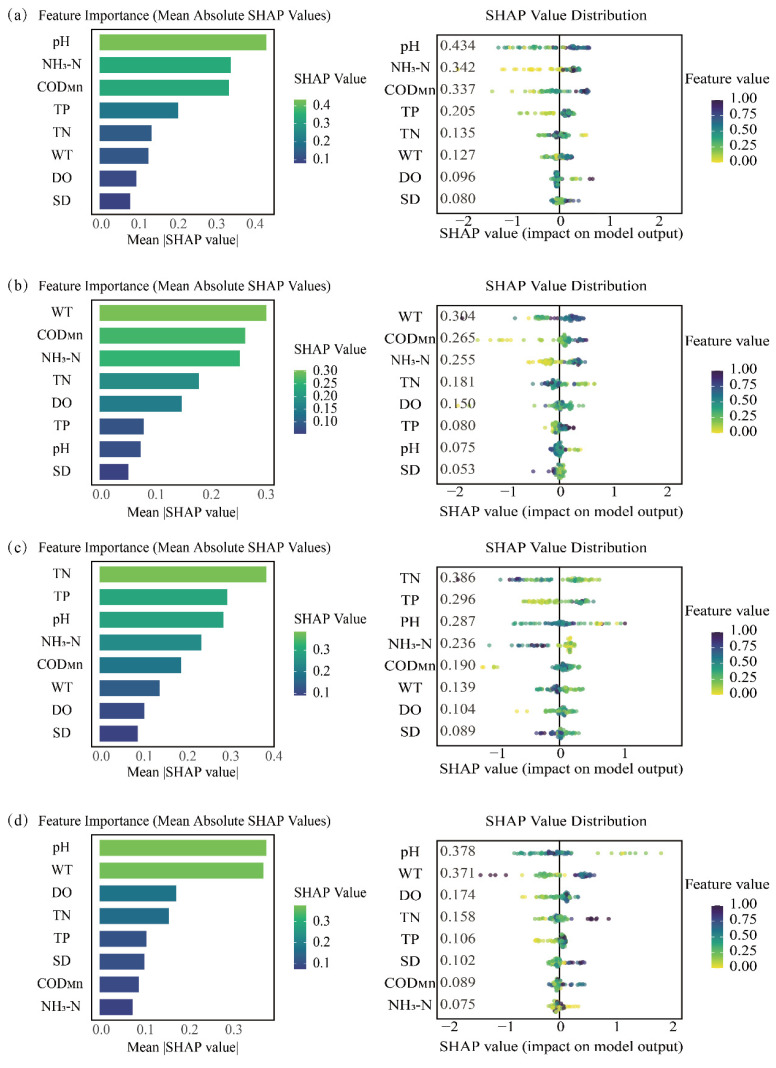
Analysis of Factor Importance and Influence Mechanisms Based on the XGBoost-SHAP Model. (**a**) Spring, (**b**) Summer, (**c**) Autumn, (**d**) Winter. A positive SHAP value indicates that the factor promotes phytoplankton abundance, while a negative value indicates suppression.

We employed the XGBoost-SHAP model, an interpretable machine learning tool, to analyze the influence of environmental factors on phytoplankton abundance in the Baihua Reservoir across different seasons. This approach quantifies and visualizes feature contributions through metrics like mean absolute SHAP value (bar plots) and the distribution of impacts (violin plots). The results indicate that the key environmental drivers influencing phytoplankton abundance exhibit marked seasonal variation (Figure 9).

**Figure 10:**
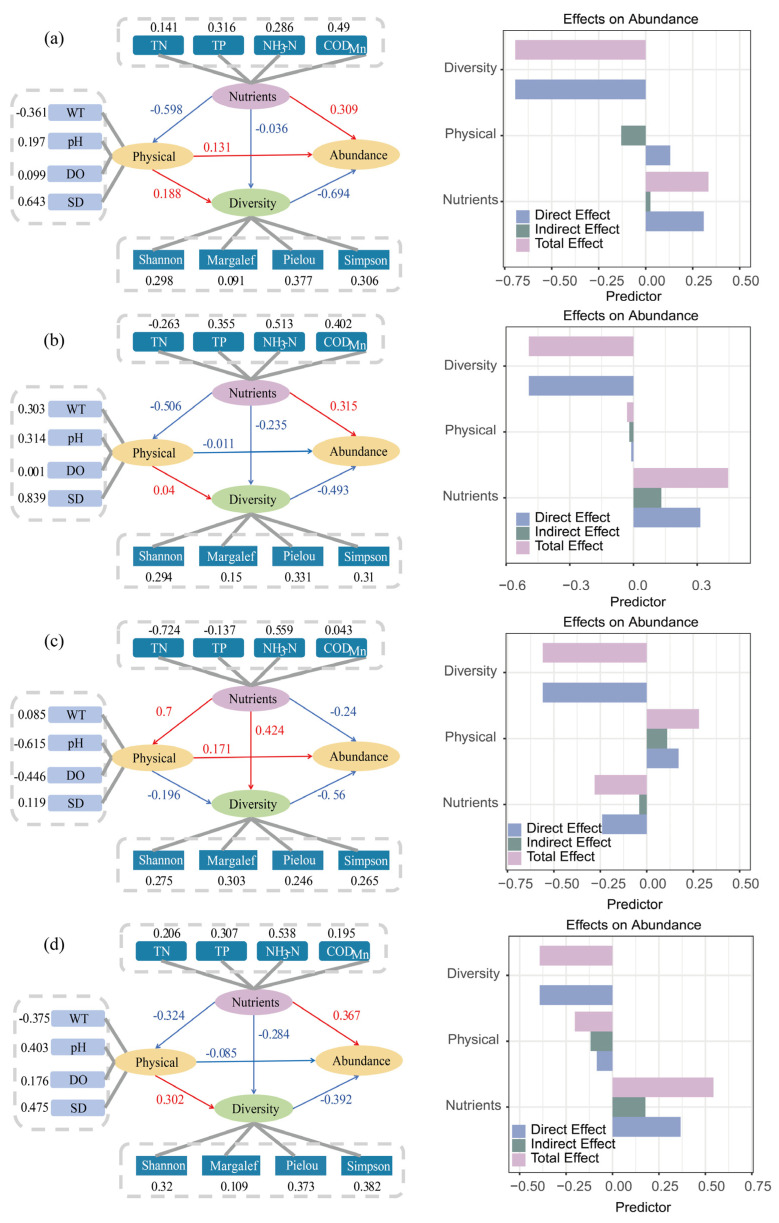
Partial Least Squares Path Model (PLS-PM) of Phytoplankton and Various Parameters: (**a**) Spring, (**b**) Summer, (**c**) Autumn, (**d**) Winter.

The primary function of Partial Least Squares Path Modeling (PLS-PM) is to quantify the direct, indirect, and total effects of physical factors (WT, pH, DO, SD), nutrients (TN, TP, NH_3_-N, COD_Mn_), and diversity indices (Shannon, Margalef, Pielou, Simpson) on phytoplankton abundance. Alpha and beta diversity indices are provided in the Supplementary Materials. The four seasonal subplots in Figure 10 clearly illustrate this complex regulatory mechanism: the path coefficients (values adjacent to the arrows) connecting each latent variable intuitively reflect the intensity and direction of influence, with positive signs representing promoting effects, negative signs indicating inhibitory effects, and absolute values closer to 1 denoting stronger effects. From a seasonal perspective, the effects of DO, WT, and pH fluctuate across seasons (DO exhibits the strongest positive effect in winter, WT in summer, and pH shows a significant positive effect in spring). Among nutrients, TP, NH_3_-N, and COD_Mn_ primarily exert positive promoting effects, serving as key drivers especially in spring and summer, while TN displays a threshold effect and shifts to negative inhibition at high concentrations. Diversity indices have a weak and directionally inconsistent direct effect on abundance, mainly exerting regulatory roles through the indirect pathway from nutrients to diversity and then to abundance. The effects of the indicators showed heterogeneity across the four seasonal subplots. Effect decomposition further clarifies the contributions of direct and indirect effects of each factor, and total effect values provide a core basis for identifying key regulatory factors. Overall, the model offers quantitative support for deciphering the multidimensional regulatory mechanisms underlying phytoplankton abundance.

## 3. Discussion

### 3.1. Phytoplankton Community Assembly

Community assembly is governed by the balance of deterministic and stochastic processes, yet the relative importance of these mechanisms remains a central debate in aquatic ecology [37,38]. Our study in Baihua Reservoir revealed that phytoplankton assembly is not dominated by a single process but rather exhibits a multi-scale hierarchical structure: stochastic processes prevail at the whole-reservoir scale, while deterministic processes drive species turnover at local microhabitat scales. The Neutral Community Model analysis showed high R^2^ values across all seasons, indicating that stochastic processes, such as random dispersal, ecological drift, and probabilistic birth-death events, play a dominant role in shaping the overall phytoplankton metacommunity [39]. This is consistent with the discovery of other large karst reservoirs in the southwest region of China. Studies in Yelang Reservoir demonstrated that stochastic assembly dominated phytoplankton community dynamics, attributed to high water exchange rates and flow-driven dispersal [40]. However, this stochastic dominance appears to be a distinctive feature of karst reservoirs compared to non-karst systems. In temperate non-karst lakes of North America, such as Lake Mendota (Wisconsin, USA), deterministic processes—particularly phosphorus-driven environmental filtering—have been shown to exert stronger control over phytoplankton assembly, with neutral processes explaining substantially less of the community variation than in karst systems [14]. This contrast highlights the unique role of hydrological connectivity in karst systems.

β-diversity decomposition provided a nuanced perspective: the consistently higher turnover component highlighted the significant role of deterministic, niche-based processes in driving species replacement. This was complemented by a measurable nestedness component, indicating additional contributions from dispersal limitation to community differences [41]. These results fundamentally reflect the multi-scale nature of community assembly. Neutral community model analysis indicated stochasticity at the whole-reservoir scale, where overall community assembly lacked significant environmental filtering or competitive exclusion and was instead dominated by random drift and dispersal [42]. In contrast, the determinism identified through β-diversity decomposition operated at the local microhabitat scale, where stable environmental heterogeneity rather than random factors selects species, leading to community differences driven by deterministic processes. At the regional scale, phytoplankton disperse and settle randomly via hydrological processes. At the local scale, environmental heterogeneity and seasonal variations filter species through niche-based processes, resulting in high species turnover. The dominance of stochastic processes at the reservoir scale is attributed to high hydrological connectivity, where water flow facilitates stochastic dispersal of phytoplankton across the study area, thereby reducing spatial heterogeneity. In contrast, microhabitat variations among sampling sites drive deterministic filtering, leading to species turnover. The seemingly divergent results thus confirm the true complexity of natural communities, which are governed not by a single mechanism but by a hybrid model combining large-scale stochasticity with small-scale determinism. This represents a core distinction between human-regulated and natural water bodies. NCM analysis of fish intestinal microbial communities across life cycle stages showed that neutral processes decline gradually with host growth and development, while deterministic selection has a more notable effect on community assembly [43]. Stochastic and deterministic processes simultaneously influence community assembly, with different methodological approaches capturing these processes from distinct perspectives [44].

The co-occurrence network characteristics varied across seasons. The spring network, with its high clustering coefficient and short average path length, indicates the formation of a tightly knit, compartmentalized community structure [45]. The exclusively positive correlations suggest harmonious interspecific relationships, likely attributable to suitable water temperatures and ample resources, which reduce competitive pressure. In contrast, the summer network exhibited lower connectivity, longer paths, and reduced clustering, signifying a complex yet loosely organized structure. The emergence of negative correlations points to intensified resource competition, potentially driven by niche partitioning under high-temperature stress. Autumn was characterized by the highest average degree, reflecting the most complex interspecific interactions. The peak in negative correlations and heightened competitive pressure imply resource limitation became a critical constraint [46]. During winter, all metrics indicated a relatively simple network structure. The disappearance of negative correlations and a return to harmonious species relationships are likely consequences of low temperatures suppressing phytoplankton growth, thereby minimizing competition. This seasonal pattern reflects a dynamic balance between environmental filtering and biological interactions: synergistic relationships dominate under favorable conditions, while competition intensifies when resources become limited [47]. The observed seasonal succession of keystone species is a direct response to environmental change that serves to stabilize the ecosystem. This stability arises from functional redundancy: as conditions shift, the ecological role of one keystone species can be supplanted by another that is better adapted, ensuring continuity of function. Consequently, the entire network of species interactions—including competition and mutualism—is reconfigured, shifting the hubs of energy flow. This dynamic reassembly enhances the community’s resilience to disturbances (e.g., rainfall, pollution, temperature shifts). Therefore, effective management must adopt a seasonally adaptive approach, aligning interventions with this predictable ecological rhythm.

### 3.2. Seasonal Regulatory Mechanisms of Multidimensional Driving Factors

RDA identified WT, SD, and TN as key drivers of phytoplankton succession. Furthermore, VPA attributed a significantly larger portion of the community variation to environmental factors (17.8%) than to spatial factors (1.2%), with a small shared effect (2.2%). This clearly demonstrates the dominant contribution of environmental filtering over spatial processes in structuring the phytoplankton community. Variance partitioning analysis showed that 78.8% of the variation in phytoplankton communities was unexplained by environmental and spatial factors, suggesting the existence of many unmeasured drivers. Unmeasured biological interactions, such as top-down control by zooplankton and benthic animals, contributed to the unexplained variation. Hydrological disturbances (water level fluctuations, flow exchange and mixing) were highly transient and stochastic, which could not be fully represented by spatial variables. A recent study on Hongfeng Reservoir in Guizhou has indicated that water level fluctuations in the reservoir indirectly enhance gross primary productivity by altering the dynamics of phytoplankton communities, highlighting the pivotal role of hydrological regulation in karst systems [48]. Unmeasured factors including micronutrients, dissolved organic matter and allelochemicals also affected phytoplankton growth and competition [49,50]. Moreover, complex nonlinear species interactions (facilitation, competition and niche differentiation) increased the unexplained variation [51,52]. Future studies combining food-web monitoring and high-frequency hydrological observations will help identify these unmeasured drivers and better reveal the mechanisms of phytoplankton community assembly.

To gain an in-depth understanding of the dynamic drivers behind phytoplankton community succession across different seasons in the Baihua Reservoir, the relative importance of various environmental factors was analyzed using the XGBoost-SHAP model. The results indicated that pH, NH_3_-N, and COD_Mn_ were the key drivers of phytoplankton abundance changes in spring. During spring, vigorous photosynthetic activity of phytoplankton consumes large amounts of CO_2_ from the water, thereby elevating the pH level. Thus, pH serves as a direct indicator of photosynthetic intensity, with higher pH values signifying that algae are in an active growth phase [53].

In spring, the vigorous growth of phytoplankton creates a substantial demand for nitrogen nutrients, making them a fundamental limiting factor for phytoplankton growth. The level of COD_Mn_ directly reflects the degree of organic pollution in the water body. Elevated COD_Mn_ concentrations indicate higher organic matter content that can be utilized by phytoplankton, thereby providing a rich nutritional foundation for their growth [54]. Consequently, COD_Mn_ serves as a critical limiting factor for phytoplankton growth and reproduction. In summer, WT, COD_Mn_, and NH_3_-N act as key drivers that co-regulate abundance. Among them, WT is particularly influential as it peaks annually. It has been well established that water temperature decisively shapes phytoplankton growth rates and species outcomes by mediating enzymatic activity and photosynthesis-respiration processes [55,56]. The predominant drivers shifted to TN, TP, and pH for phytoplankton abundance in autumn. The seasonal decline in temperature and light triggers a shift in the primary control of phytoplankton growth from physical drivers to the absolute supply of nutrients, with TN and TP emerging as the most significant factors [57]. In winter, pH, WT, and DO serve as the key drivers, collectively representing the ambient physicochemical background. This signifies a period when the biological influence of phytoplankton is minimal, and baseline environmental conditions defined by low temperature and weak photosynthesis become the dominant controlling factors [58]. Overall, the key environmental drivers governing phytoplankton succession in Baihua Reservoir exhibit marked seasonal variations. This reflects a fundamental shift in primary constraints: from pH and NH_3_-N in spring, to elevated temperature and COD_Mn_ in summer, followed by TN and TP co-limitation in autumn, and finally to pH combined with low temperature in winter.

### 3.3. Implications for Ecosystem Management

Based on a comprehensive study of the phytoplankton community assembly mechanisms and environmental driving factors in Baihua Reservoir, this paper proposes the following systematic management strategy. The overall approach should integrate both deterministic environmental filtering and stochastic dispersal processes, focusing not only on controlling phytoplankton abundance but also on maintaining community structure stability. Specifically, seasonally differentiated measures are recommended:

In spring, priority should be given to controlling organic pollutant inputs to reduce COD_Mn_ and stabilize pH; in summer, nutrient management should be intensified, with particular emphasis on phosphorus load reduction and cyanobacterial bloom prevention under high-temperature conditions; in autumn, efforts should focus on maintaining nitrogen-phosphorus balance to prevent structural imbalances in nutrients that may lead to community simplification; in winter, it is essential to preserve baseline water quality stability and hydrological conditions to support community recovery.

The findings indicate that management efforts must center on reducing nitrogen, phosphorus, and COD_Mn_ levels while elevating DO as a key mitigation strategy. Compared to non-karst reservoirs, the high carbonate buffering capacity of Baihua Reservoir could also be the reason why pH becomes a key driver; thus, its management requires additional attention to pH regulation. It is also essential to preserve hydrological connectivity, minimize artificial barriers, and reduce water-level fluctuations to alleviate the adverse effects of hydrological disturbances on ecological processes. To improve long-term management efficacy, future efforts should incorporate monitoring of heavy metal concentrations and zooplankton composition, enabling the development of a comprehensive eco-environmental response database. Furthermore, an early-warning platform integrating key parameters for algal bloom risk assessment should be established to shift from reactive interventions to proactive regulation.

In summary, the effective management of Baihua Reservoir constitutes a comprehensive, ecosystem-based engineering approach. Given its high carbonate buffering capacity (pH 7.39–8.72), the reservoir requires that pH be regulated in concert with TN, TP, and COD_Mn_. This strategy, therefore, focuses on the control of these key parameters and is grounded in maintaining hydrological connectivity and habitat stability, while being dynamically optimized in response to seasonal variations.

## 4. Materials and Methods

### 4.1. Study Area

**Figure 11:**
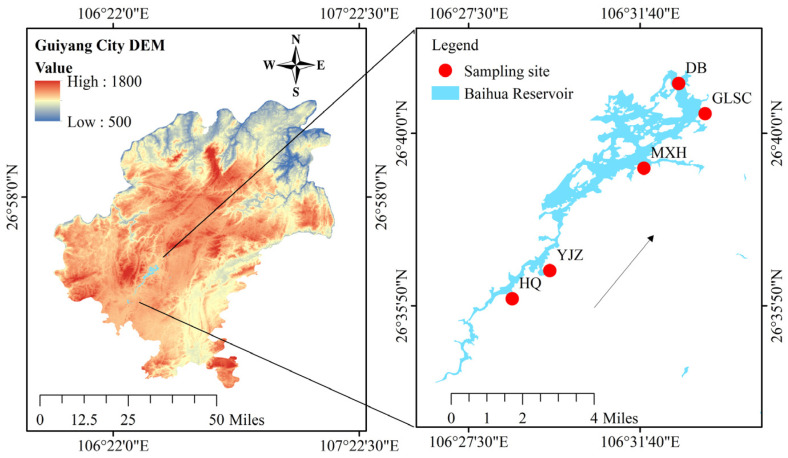
Map of the study area in Baihua Reservoir showing the sampling locations. Arrows indicate water flow direction.

Baihua Reservoir (26°35′~26°41′ N, 106°27′~106°32′ E) is a strategically important water body situated in the karst region of southwestern China. It forms part of the Maotiao River basin within the Wujiang River system, which is itself a major tributary of the Yangtze River (Figure 11). The reservoir serves multiple critical functions, including acting as a crucial drinking water source for Guiyang City, alongside providing hydropower generation, flood control, agricultural irrigation, and supporting tourism activities. Hydrologically, it features a basin area of 1895 km^2^, a total storage capacity of 1.82 × 10^8^ m^3^, and an annual water supply volume of 2.87 × 10^7^ m^3^. Its morphometry is characterized by a maximum depth of 45 m and an average depth of 10.8 m, classifying it as an elongated, typical karst reservoir in Guizhou Province. The reservoir shore is characterized by numerous rural settlements and agritourism facilities, making its water quality highly susceptible to anthropogenic activities and intrinsically linking it to the health of the local aquatic ecosystem and socio-economic well-being. To represent its hydrological gradient, five sampling sites were strategically established from the head to the tail: Huaqiao (HQ), Yanjiaozhai (YJZ), Maixihe (MXH), Guilvshuichang (GLSC), and Daba (DB).

### 4.2. Samples Collection and Analysis

Monthly sampling of water and phytoplankton was conducted at five sites in BHR from January 2020 to December 2024. Water temperature (WT), dissolved oxygen (DO), and pH were measured on-site using a portable multi-parameter water quality analyzer (HANNA HI 98194, Shenzhen, China), while transparency (SD) was determined with a Secchi disk.

Water samples (3 L) were collected at each site using a 5 L water sampler and subdivided into two 1.5 L polyethylene bottles. One subsample was designated for phytoplankton analysis and was immediately preserved with 1.5% Lugol’s solution. In the laboratory, these preserved samples were sedimented for 24–48 h. Following sedimentation, the supernatant was carefully siphoned off, resulting in a concentrated sample with a final volume of 30 mL. Immediately following concentration, each sample was labeled and accompanied by metadata detailing its collection time, location, and pre- and post-concentration volumes.

Taxonomic identification and enumeration were performed using a biological microscope (Olympus CX43, Shanghai, China). Morphological identification of phytoplankton was primarily based on *Freshwater Algae of China: Systematics, Taxonomy, and Ecology*, a comprehensive reference widely used for freshwater algae in China [59]. To ensure taxonomic accuracy and consistency with current nomenclatural standards, all identified species were cross-checked against the AlgaeBase database (www.algaebase.org) [60]. Before counting, the samples were homogenized through gentle agitation. A 100 μL aliquot was transferred to a Sedgwick-Rafter counting chamber, covered with a coverslip to eliminate air bubbles, and examined at 400× magnification using the ocular grid method. Phytoplankton abundance (cells/L) was calculated based on taxon counts and standardized to the original sample volume. Additionally, a separate 1.5 L sample was analyzed to determine total nitrogen (TN), total phosphorus (TP), permanganate index (COD_Mn_), and ammonia nitrogen (NH_3_–N) in accordance with Chinese national standard methods for water quality testing [61].

### 4.3. Data Analysis

All statistical analyses were performed using SPSS Statistics 26 and R (version 4.5.1). Data visualization was conducted using OriginPro 2025 (OriginLab, Northampton, MA, USA) and Gephi 0.10.

#### 4.3.1. Determination of Dominant Species

The McNaughton dominance index (Y) was employed to identify dominant phytoplankton species, calculated using the formula Y = (Ni/N) · fi, where Ni is the abundance of species i; N represents the total abundance of all species; fi is the occurrence frequency of the species in each sampling site; the phytoplankton species with Y ≥ 0.02 are deemed the dominant species, and when Y > 0.1, they are the absolute dominant species.

#### 4.3.2. Seasonal Differences in Environmental Variables

One-way analysis of variance (ANOVA) was employed to test for significant differences in water physicochemical parameters across seasons. When a significant overall effect was detected (*p* < 0.05), the Least Significant Difference (LSD) post hoc test was applied for pairwise comparisons between seasons.

#### 4.3.3. Multivariate Statistics and Mechanistic Modeling

A suite of multivariate analyses and ecological models was implemented in R to elucidate phytoplankton community dynamics. Redundancy Analysis (RDA) combined with Variation Partitioning Analysis (VPA) was used to quantify the relative contributions of environmental and spatial factors to community variation. The Neutral Community Model (NCM) was fitted to assess the role of stochastic processes in community assembly, following the approach of Sloan et al. [62]. β-diversity was quantified using Sørensen and Jaccard indices and further decomposed into turnover and nestedness components to distinguish between deterministic and stochastic assembly processes [63]. Partial Least Squares Path Modeling (PLS-PM) was applied to evaluate the direct and indirect effects of physical factors, nutrients, and diversity indices on phytoplankton abundance.

#### 4.3.4. Machine Learning and Variable Importance

To assess the relative importance of environmental variables in shaping phytoplankton abundance, the XGBoost machine learning algorithm was applied. Model interpretability was enhanced by computing SHapley Additive exPlanations (SHAP) values using the ‘SHAP’ package in R4.5.3. Variable importance was ranked based on mean absolute SHAP values, with higher values indicating greater influence on phytoplankton dynamics.

#### 4.3.5. Co-Occurrence Network Analysis

Co-occurrence networks were constructed based on Spearman correlation matrices calculated in R. Significant correlations were retained and visualized using Gephi software. Network topology parameters—including number of nodes and edges, average degree, average path length, and clustering coefficient—were computed to characterize community complexity, connectivity, and modularity.

## 5. Conclusions

Based on this study, the dynamics of phytoplankton in Baihua Reservoir are governed by three interconnected core mechanisms: multidimensional environmental drivers exhibit significant seasonal variations; both deterministic and stochastic processes collectively shape community assembly; and interaction network patterns validate the complexity of their synergistic effects. Therefore, the following targeted control strategies are proposed: TN, TP, and COD_Mn_ should be prioritized as key pollutants for reduction, while DO levels need to be consistently maintained and enhanced, with pH undergoing monitored regulation. At the management level, it is essential to preserve hydrological connectivity and minimize hydrological disturbances to ensure the integrity of ecological processes. Additionally, seasonally differentiated management measures should be established to achieve effective regulation of the phytoplankton community and support the long-term health of the reservoir ecosystem.

## Figures and Tables

**Table 1:** Topological parameters of the phytoplankton co-occurrence network in Baihua Reservoir.

Topological Parameter	Spring	Summer	Autumn	Winter
nodes	23.00	37.00	40.00	40.00
edges	20.00	36.00	42.00	32.00
positive correlation	20.00	33.00	36.00	32.00
negative correlation	0.00	3.00	6.00	0.00
average degree	1.74	1.95	2.10	1.60
average path length	0.97	2.11	1.50	1.11
network diameter	2.67	5.09	3.89	2.60
network density	0.08	0.05	0.05	0.04
clustering coefficient	0.60	0.33	0.35	0.47

## Data Availability

The original contributions presented in this study are included in the article/Supplementary Material. Further inquiries can be directed to the corresponding authors.

## Supplementary Materials

The following supporting information can be downloaded at https://www.mdpi.com/article/10.3390/plants15071024/s1, Figure S1: Seasonal alpha diversity changes of phytoplankton in Baihua Reservoir; Figure S2: Seasonal beta diversity changes of phytoplankton in Baihua Reservoir.
