# Supplementary material for: Multidimensional Drivers of Phytoplankton Assembly in a Karst Reservoir: Seasonal Dynamics and Regulatory Implications
Source: Plants (Basel). 2026 Mar 26;15(7):1024. doi: 10.3390/plants15071024 (PMC13074921; doi:10.3390/plants15071024)
Supplement: Supplementary file 1 [file plants-15-01024-s001.zip › plants-4215114-supplementary.pdf]

## Supplementary Materials

The analysis of α-diversity across seasons indicated that the Margalef richness index was the only metric that showed a significant difference, which was specifically observed between spring and summer (Figure S1).

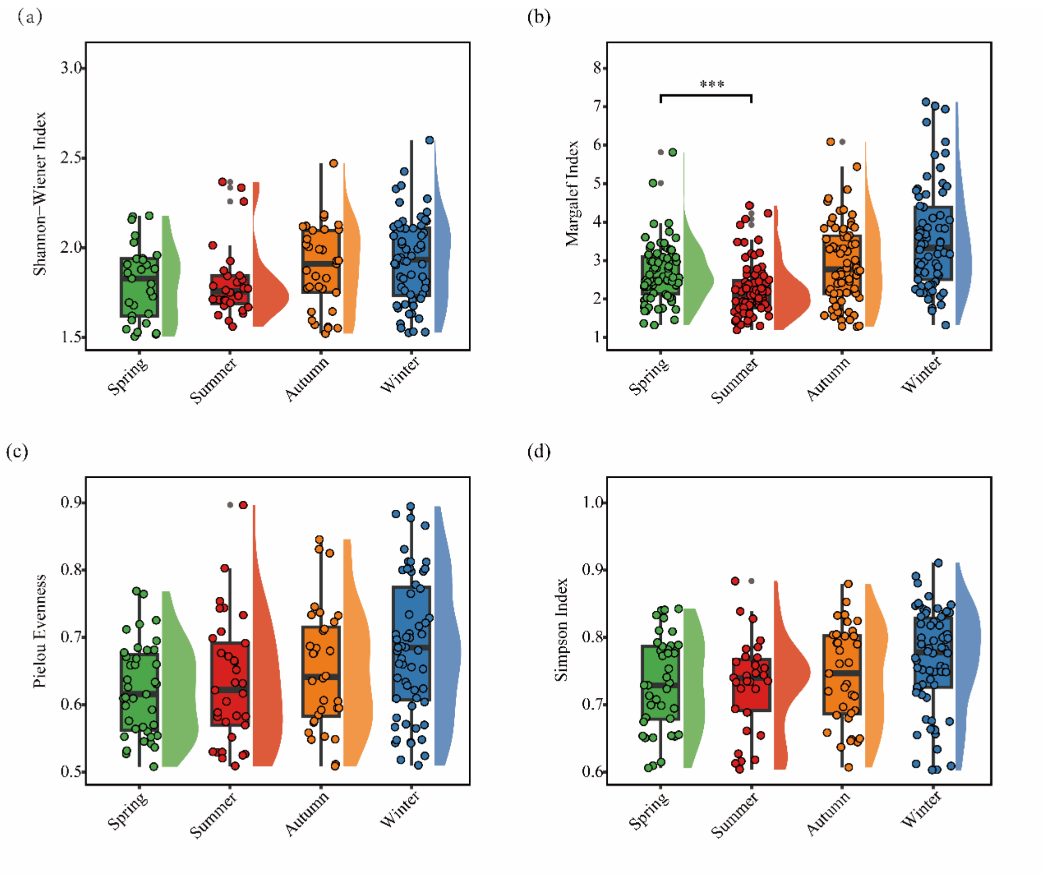


**Figure S1.** Seasonal alpha diversity changes of phytoplankton in Baihua Reservoir

The β-diversity of the phytoplankton community exhibited significant seasonal dissimilarity, as determined by ANOSIM and visualized in the NMDS plot (Figure S2)

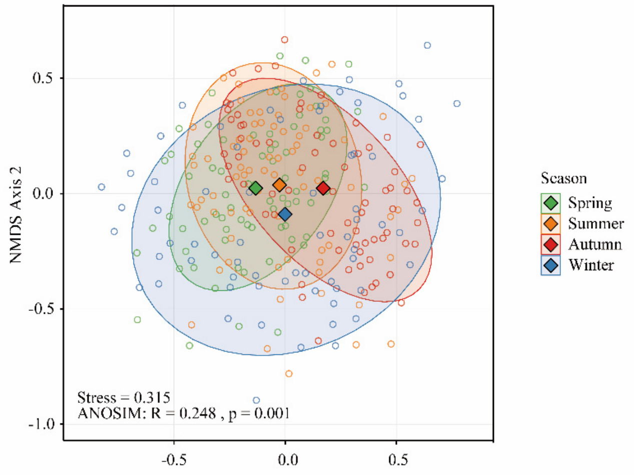


**Figure S2.** Seasonal beta diversity changes of phytoplankton in Baihua Reservoir
